# Multiplexing polysome profiling experiments to study translation in *Escherichia coli*

**DOI:** 10.1371/journal.pone.0212297

**Published:** 2019-02-19

**Authors:** Huong Le Nguyen, Marie-Pierre Duviau, Muriel Cocaign-Bousquet, Sébastien Nouaille, Laurence Girbal

**Affiliations:** LISBP, Université de Toulouse, CNRS, INRA, INSA, Toulouse, France; John Curtin School of Medical Research, AUSTRALIA

## Abstract

Polysome profiling is a widely used method to monitor the translation status of mRNAs. Although it is theoretically a simple technique, it is labor intensive. Repetitive polysome fractionation rapidly generates a large number of samples to be handled in the downstream processes of protein elimination, RNA extraction and quantification. Here, we propose a multiplex polysome profiling experiment in which distinct cellular extracts are pooled before loading on the sucrose gradient for fractionation. We used the multiplexing method to study translation in *E*. *coli*. Multiplexing polysome profiling experiments provided similar mRNA translation status to that obtained with the non-multiplex method with comparable distribution of mRNA copies between the polysome profiling fractions, similar ribosome occupancy and ribosome density. The multiplexing method was used for parallel characterization of gene translational responses to changing mRNA levels. When the mRNA level of two native genes, *cysZ* and *lacZ* was increased by transcription induction, their global translational response was similar, with a higher ribosome load leading to increased ribosome occupancy and ribosome densities. However the pattern and the magnitude of the translational response were gene specific. By reducing the number of polysome profiling experiments, the multiplexing method saved time and effort and reduced cost and technical bias. This method would be useful to study the translational effect of mRNA sequence-dependent parameters that often require testing multiple samples and conditions in parallel.

## Introduction

In the last decade, interest in the role of regulating translation has been increasing. Translation regulation plays an essential role in fine-tuning gene expression and protein level [1]. It allows a rapid response to extracellular stimuli, which can be crucial for adaptation to different environmental conditions [2]. Polysome profiling is a widely used method to study translation status. For each individual gene, the method quantifies the number of ribosomes bound to each copy of the mRNA molecule and provides a detailed distribution of the mRNA copies per number of bound ribosomes (proportions of free mRNA copies, of monosome-bound and polysome-bound mRNA copies [3–5]. Polysome profiling enables the definition of two translational variables, ribosome occupancy (RO) and ribosome density (RD) [6–8]. RO is the proportion of mRNA copies bound with at least one ribosome and can be used as a proxy for translation initiation. RD can be calculated as the number of mRNA-bound ribosomes in each polysome profiling fraction normalized to the coding sequence length; it reflects the level of translation initiation, elongation and termination. Thus, polysome profiling not only assesses heterogeneity in the number of bound ribosomes within the copies of an mRNA, but also accesses physical ribosome density by measuring joint binding of multiple ribosomes on the same transcript. This technique is complementary to the recently expanding method of ribosome profiling that quantifies the heterogeneity of ribosome position occupation averaged over the population of mRNA copies.

The polysome profiling method can be used to study translational status in different organisms at different stages of growth and development (e.g. *Arabidopsis thaliana* [9], in sea urchin [10], in halophilic archaea [3]). It is also used to understand translational response to various stresses: marine organisms under oxidative stress [11], yeast and *A*. *thaliana* in high salinity conditions [12,13], yeast under Zn limitation [14] and *Lactococcus lactis* and yeast under nutrient starvation [8,15]. It is also frequently used in mechanistic studies of translation regulation, for instance to characterize the effects of elongation factors [16,17]. The role of mRNA sequence related parameters such as 5’UTR and codon usage on translation has also been investigated using polysome profiling [18–20].

These studies are usually limited to a small number of samples and conditions because polysome profiling is labor intensive. The drawbacks of the technique remain the main difficulty in handling many samples in parallel [21]. Separation of mRNA-polysome complexes according to bound ribosomal loading consists in polysome fractionation on the sucrose gradient. This step generates numerous samples to be handled in the downstream processes of protein elimination, RNA extraction and quantification. This may introduce technical bias between different polysome profiling experiments and also entails rather expensive and time consuming experiments. We developed a multiplexing method for polysome profiling experiments that makes it possible to assemble six different cell free extracts before loading on the sucrose gradient. The RNA in each fraction was quantified by RT-qPCR with an optimized amount of exogenous RNA spike-ins. A challenge in multiplexing experiment is to differentiate the cell free extract origin of the measured mRNAs. In this study, we identified the origin of an mRNA of interest by strongly overexpressing this mRNA in only one cell free extract of the mixture. The multiplexing polysome profiling method was then used to study translation regulation in the bacterial model *Escherichia coli* K12 MG1655. The translational states of different genes were simultaneously characterized by measuring their ribosome occupancies and ribosome densities. We also accessed the translational response of these genes to changing gene expression, a situation that may be encountered when *E*. *coli* cells need to adapt to variable growth environments.

## Materials and methods

### Plasmids and strain construction

All strains were constructed in the genetic background *E*. *coli* MG1655 Δ*araFGH*, Ω*pcp18*::*araE533* [22]. MET734 and MET739 carried the *cysZ* and *lacZ* genes, respectively, on a plasmid under the P_BAD_ inducible promoter (Table 1). We selected these two native genes of *E*. *coli* MG1655 for their low level of expression in exponential growth in synthetic medium [23]. For each, the 5’UTR + ORF fragment was amplified by PCR and cloned into the pBAD/myc/His plasmid (Invitrogen) to obtain the constructs: pBAD– 5’UTR+ORF_selected gene_—myc/His tag. The pBAD-5’UTR+ORF_lacZ_-myc/His tag was introduced into the *E*. *coli* variant where the chromosomal copy of *lacZ* was deleted [24]. Four other “filling” strains were used to mix their cell free extract with those of MET734 and MET739 for multiplexing purposes. In the same genetic background, the four strains contained genes (*yeeZ*, *inaA*, *ucpA* and *yjc*) not related to this study, under the control of P_BAD_.

**Table 1:** Strain description.

Strain	Description	Source
DCT2202	*E*. *coli* MG1655 Δ*araFGH*, Ω*pcp18*::*araE533*	[22]
MET345	DCT2202 Δ*lacZ*	[24]
MET739	MET345 with plasmid (pBAD-lacZ-*myc*-his)	This work
MET734	*E*. *coli* DCT2202 with plasmid (pBAD-cysZ-*myc*-his)	This work

### Culture and preparation of cell lysate

Each strain was individually grown in chemically defined minimum medium M9 supplemented with 3 g/L glucose [23], 0.1 mg/mL ampicillin, at 37 °C, under shaking (150 rpm). Arabinose was added at a final concentration of 0.001% (w/v) when the culture reached an OD_600_ of 1 (exponential growth). After 30 minutes of induced gene expression, chloramphenicol was added at a final concentration of 0.1 mg/mL to stop translation elongation.

Cell culture was immediately transferred on ice for one minute. A fixed volume corresponding to 320 ml per OD unit was collected and centrifuged at 6,300 g, at 4 °C for 15 minutes. The supernatant was discarded and the cell pellet washed twice with cold lysis buffer (20 mM Tris HCl pH 8, 140 mM KCl, 40 mM MgCl_2_, 0.5 mM DTT, 100 μg/mL chloramphenicol, 1 mg/mL heparin, 20 mM EGTA, 1% (v/v) Triton X-100) and resuspended in 1.2 mL of cold lysis buffer.

The cell suspension was transferred in cold screw-capped tubes containing 0.1 g of glass beads (0.1 mm diameter, Sigma) and disrupted using a FastPrep-24 (MP Biomedicals). We observed that more RNA was obtained when we performed four 30 s cycles at 6.5 m/s with at least one minute on ice between cycles rather than two cycles. The lysate was first centrifuged for 5 minutes at 2,100 g at 4 °C to remove the glass beads. The supernatant was collected and centrifuged again for 5 minutes at 8,600 g, at 4 °C. Clarified lysate was gently collected, immediately frozen in liquid nitrogen and stored at -80 °C. The concentration of protein in the lysate was measured using a NanoDrop ND-1000 UV spectrophotometer (Nanodrop Technologies). Starting from around 110 mg of dry cell weight, the protein yield was around 50–60 mg/mL. All the steps were performed at 4 °C and the samples were kept on ice.

### Polysome profiling experiments

According to ribosome loading, the mRNA-ribosome complexes were separated on 10–50% (w/v) linear sucrose gradient (24 mL) prepared in cold lysis buffer. In the non- multiplexing experiment, individual cell free extract (≈ 2.4 mL) was loaded on the sucrose gradient, whereas in the multiplexing experiment, six cell free extracts were pooled to reach an equivalent protein amount per strain in a final volume of 2.4 mL. Ultracentrifugation was performed in a Sorvall WX80 (ThermoScientific) using a swing rotor AH-629, for 16h30min, at 23,700 g, at 4 °C. The sucrose gradient was eluted with cold buffer (55% sucrose (w/v), 0.5 mM Tris HCl pH 8, 4 μg/mL Bromophenol blue) in 24 sub-fractions at 2.5 mL/minute. Absorbance was continuously measured at 254 nm with a UV detector (UPC900 Amersham Pharmacia Biotech).

### Protein elimination and RNA extraction

Protein denaturation and nucleic acid precipitation were performed by adding one volume of 8 M guanidium-HCl, two volumes of absolute ethanol and overnight storage at -20°C. After 30 minutes centrifugation at 13,300 g at 4 °C, the supernatant containing the free proteins was gently removed and the pellet of nucleic acids (including mRNA loaded with ribosomes) were washed with cold 75% ethanol (v/v) and resuspended in TE buffer (10 mM Tris HCl pH 8, 1 mM EDTA). Total RNA extraction and purification were performed using the extraction RNeasy Midi kit (Qiagen). Genomic DNA was removed by on-column DNase digestion using 90U of RNase-free DNase I (Qiagen) for 15 minutes at room temperature. The total RNA concentration was measured using NanoDrop ND-1000. RNA integrity was validated and 16S and 23S rRNA were quantified using Bioanalyzer 2100 (Agilent Technologies). Total RNA samples were stored at -80 °C.

### Reverse transcription and RNA quantification by real-time quantitative PCR

Total RNA (5 μg) was reverse-transcribed to yield cDNA using 200U of SuperScript II reverse transcriptase (Invitrogen) as previously described [25]. cDNA was quantified by Real Time PCR Detection system (Bio-Rad) in 96-well plates as previously described [26]. cDNA dilutions of 10^−1^ and 10^−2^ were used to provide quantifiable signals, i.e. cycle threshold (Ct) of between 15 and 25. For large numbers of samples, a high-throughput qPCR technique was applied using Biomark HD System (Fluidigm Corporation, CA, USA) as previously described [24].

To account for variability of the reverse transcription and the qPCR steps between samples and experiments, control Ambion ERCC RNA Spike-In mix was used as external normalizer. For each fraction, an equal amount of ERCC was added to a constant amount of total RNA and they were then reverse transcribed together. To improve the efficiency and reproducibility of the reverse transcription of ERCC, 0.2 μM of reverse primers specific to the four most concentrated ERCCs (ERCC 130, ERCC 002, ERCC 074 and ERCC 096) were added during reverse transcription in addition to random primers.

A total of 12 different mRNAs and four ERCCs were quantified in this work (S1 Table). Quantification of *lacZ* mRNA is the average value obtained from five primer pairs. Primers for qPCR were designed for these 20 genes using Vector NTI advance v11 (Life Technologies) using a melting temperature of 59–61 °C, length of 18–20 bp and 50–70% GC content (S1 Table). Amplicon sizes ranged between 80 and 150 bp. The reaction efficiency was tested on cDNA serial dilutions and focused around 100%.

### Data normalization and analysis

To calculate the relative amount of a target mRNA in each fraction, two normalizations were applied. First, relative mRNA abundance compared to a constant quantity of ERCC was calculated using the method of fold change ΔCt values [27]. As only 5 μg of the total RNA amount extracted in each fraction was used in the RT-qPCR experiment, we normalized the relative mRNA abundance by the total RNA quantity extracted in each fraction to obtain the relative initial mRNA abundance in each fraction.

For each target gene, its relative initial mRNA abundance compared to ERCC in fraction *i* was calculated as follows:

$$Relative\;initial\;mRNA\;abundace\;=\;2^{(Ct\;ERCC_{i}-Ct\;target_{i\;})\;}*\;\frac{total\;RNA\;quantity_{i}\;}{5}$$

To obtain the distribution of the abundance of mRNA copies, the proportion of mRNA copies in each fraction was calculated by dividing the relative initial abundance in one fraction by the sum of the abundances in all the fractions:

$$Proportion\;in\;fraction\;i\;=\;\frac{Relative\;initial\;abundance\;in\;fraction\;i}{\sum_{j\;=\;1}^{7}Relative\;initial\;abundance\;in\;fraction\;j}*100$$

### Calculation of ribosome occupancy and ribosome density

**Fig 1:**
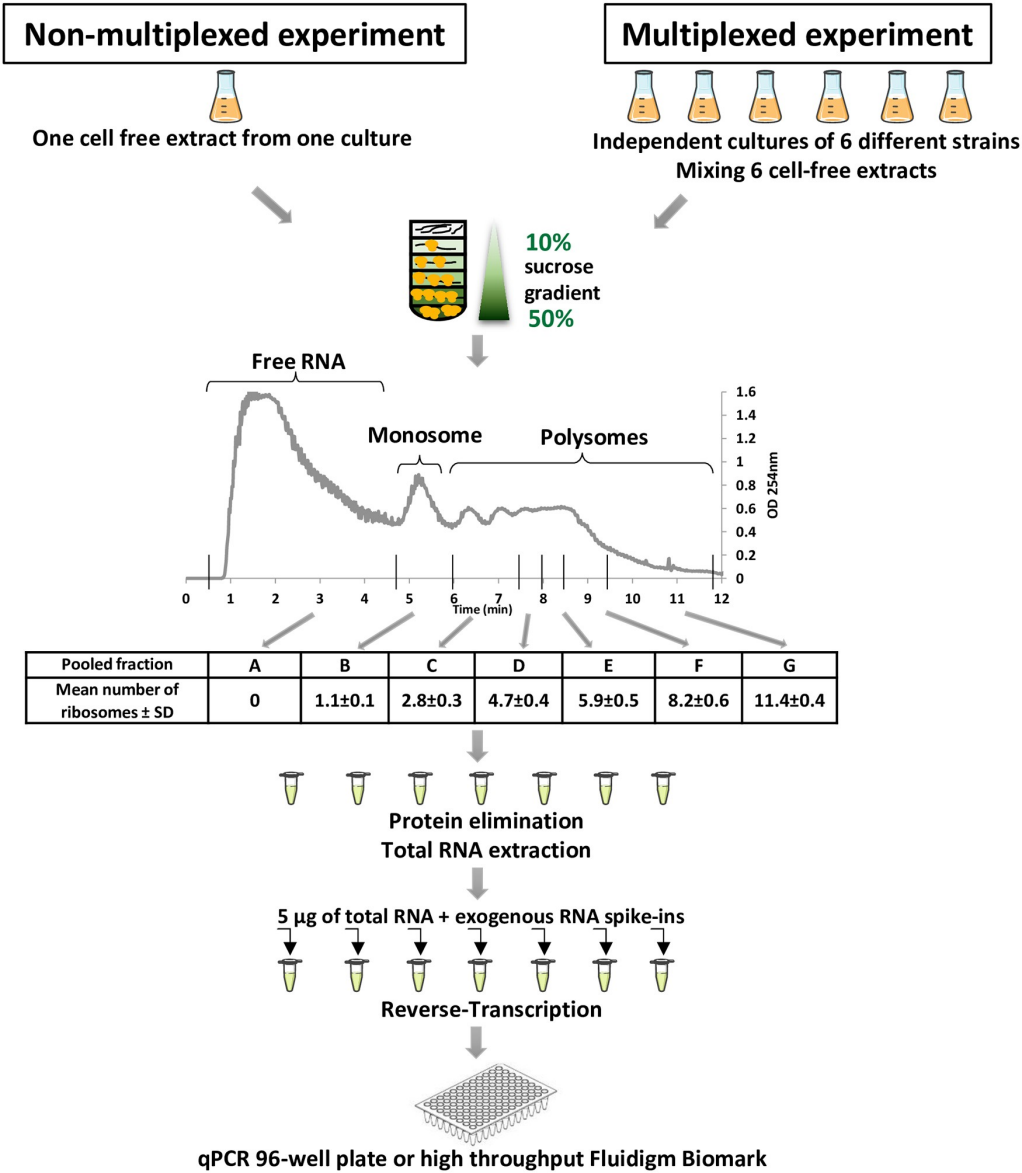
Polysome profiling experiment at a glance. All cultures and polysome profiling experiments were repeated twice to provide independent biological replicates.

For each gene, the ribosome occupancy was the proportion of the mRNA copies undergoing translation. It was calculated by summing the proportions of mRNA copies in fractions containing mRNAs bound to at least one ribosome (from fractions B to G in Fig 1). For each gene, we calculated the ribosome density in each fraction as the number of ribosomes bound to the mRNA copies normalized with respect to the coding sequence length.

## Results

### Polysome profiling experiment and translational parameters

The polysome profiling method was applied to *E*. *coli* MET739 cells during exponential growth on glucose in M9 minimum medium (Fig 1). Translation elongation was stopped by adding chloramphenicol and the cells were washed and lysed. The cell free extract was loaded on a sucrose gradient to fractionate the mRNA-ribosome complexes according to the number of bound ribosomes. A representative polysome profile is shown in Fig 1. The polysome profile was eluted in a total of 24 sub-fractions. After protein elimination and total RNA extraction, peaks were assigned by estimating the ratios between the 23S rRNA and 16S rRNA in each sub-fraction (S1 Fig). Once the components of each sub-fraction were identified, sub-fractions were grouped in seven fractions from A to G (Fig 1). Fraction A comprised sub-fractions containing DNA, free RNAs and the small and large ribosomal subunits. The two small and large ribosomal subunits were respectively identified through the high 16S/23S rRNA and 23S/16S rRNA ratios (S1 Fig). In fractions B to G, the 23S/16S rRNA ratios were constant around 1.8 and matched entire ribosomes. The 2^nd^ peak (fraction B) was attributed to the monosome. The 3^rd^ and 4^th^ peaks corresponding to two and three ribosomes were grouped in fraction C. The 5^th^ peak corresponded to four ribosomes. The number of ribosomes in the following fractions was extrapolated as previously described [7]. The mean value of the number of bound ribosomes in fractions B to G was calculated from four independent experiments (Fig 1). We chose to exemplify the estimations of the translational parameters RO and RD using the *ihfB* gene. It is a well-expressed gene in *E*. *coli* coding for an integration host factor β-subunit commonly used as an internal normalization control in RT-qPCR experiments [24,28]. To estimate *ihfB* RO and RD, we quantified the abundance of *ihfB* mRNA copies in fractions A to G.

**Fig 2:**
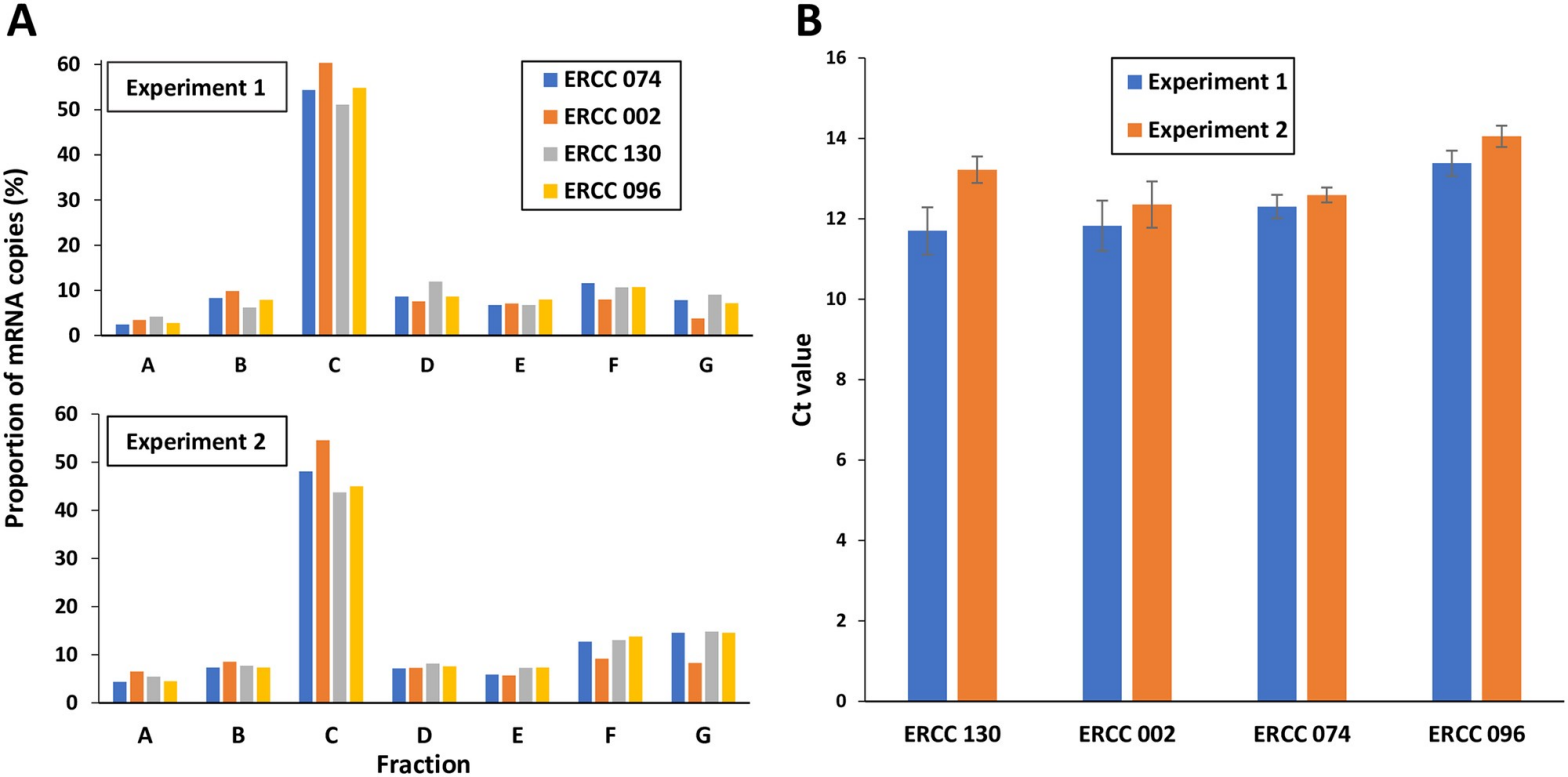
Distribution of *ihfB* mRNA copies in polysome fractions. (A) Proportion of *ihfB* mRNA copies in fractions A to G in two independent polysome profiling experiments from MET739 cell free extract when normalized by the four most concentrated ERCC (ERCC 130, ERCC 002, ERCC 074 and ERCC 096) using an RNA/ERCC ratio of 5/0.001 in μg/μL. (B) Variations in the levels of ERCC 130, ERCC 002, ERCC 074 and ERCC 096 estimated in two independent experiments. Mean values and standard deviations were calculated using the level values in the seven fractions (from A to G) of the same experiment.

A first normalization of *ihfB* mRNA abundance was performed using the ERCC RNA spike-ins. The ERCC RNA spike-ins consisted in a mix of 92 transcripts with a wide range of lengths, GC contents and concentrations. A constant quantity of the ERCC RNA spike-in mix was introduced in all the total RNA samples before the reverse transcription step. Different total RNA/ERCC ratios (in μg/μL) were tested: 2.5/0.01, 4/0.01, 5/0.01, 2/0.02, 5/0.03 and the very high ratio of 5/0.001. For the 5/0.001 ratio, oligonucleotides specific to the ERCC RNA spike-ins were added to increase their reverse transcription. The highest reverse transcription efficiencies of ERCC and RNA were obtained with the very high ratio of 5/0.001. This ratio was thus chosen for all the ERCC normalization steps. The *ihfB* mRNA abundance was then normalized by the abundance of the four most concentrated ERCCs (ERCC 130, ERCC 002, ERCC 074 and ERCC 096) and by the initial mRNA abundance in each fraction to provide the relative initial *ihfB* mRNA abundance in each fractions. The distributions of *ihfB* mRNA copy abundances between fractions A and G were then calculated to provide the typical plots of translational status (Fig 2A). Normalizations using any of the four ERCC led to similar distribution of *ihfB* mRNA copies between the polysome fractions, so any of the four ERCC can be used to analyze a translational status. For further analyses, ERCC 074 (522pb, 35% GC, 15x10^-21^ mole/μL) was selected as normalizing ERCC, as it displayed the smallest variability between fractions and experiments (Fig 2B). The translational status of *ihfB* was characterized by an RO of 96.6 ± 0.4% corresponding to 3.4 ± 0.4% of ribosome-free mRNA copies not undergoing translation. Half the *ihfB* mRNA copies (in fraction C) were loaded with around 2.8 ribosomes corresponding to a ribosome density of 1 ribosome/100 nt and around 22% of the *ihfB* mRNA copies were heavily-loaded (in fractions F and G) with more than 8.2 bound ribosomes corresponding to a RD higher than 2.9 ribosomes/100 nt.

### Multiplexing polysome profiling experiment does not alter the translational status of an mRNA

**Fig 3:**
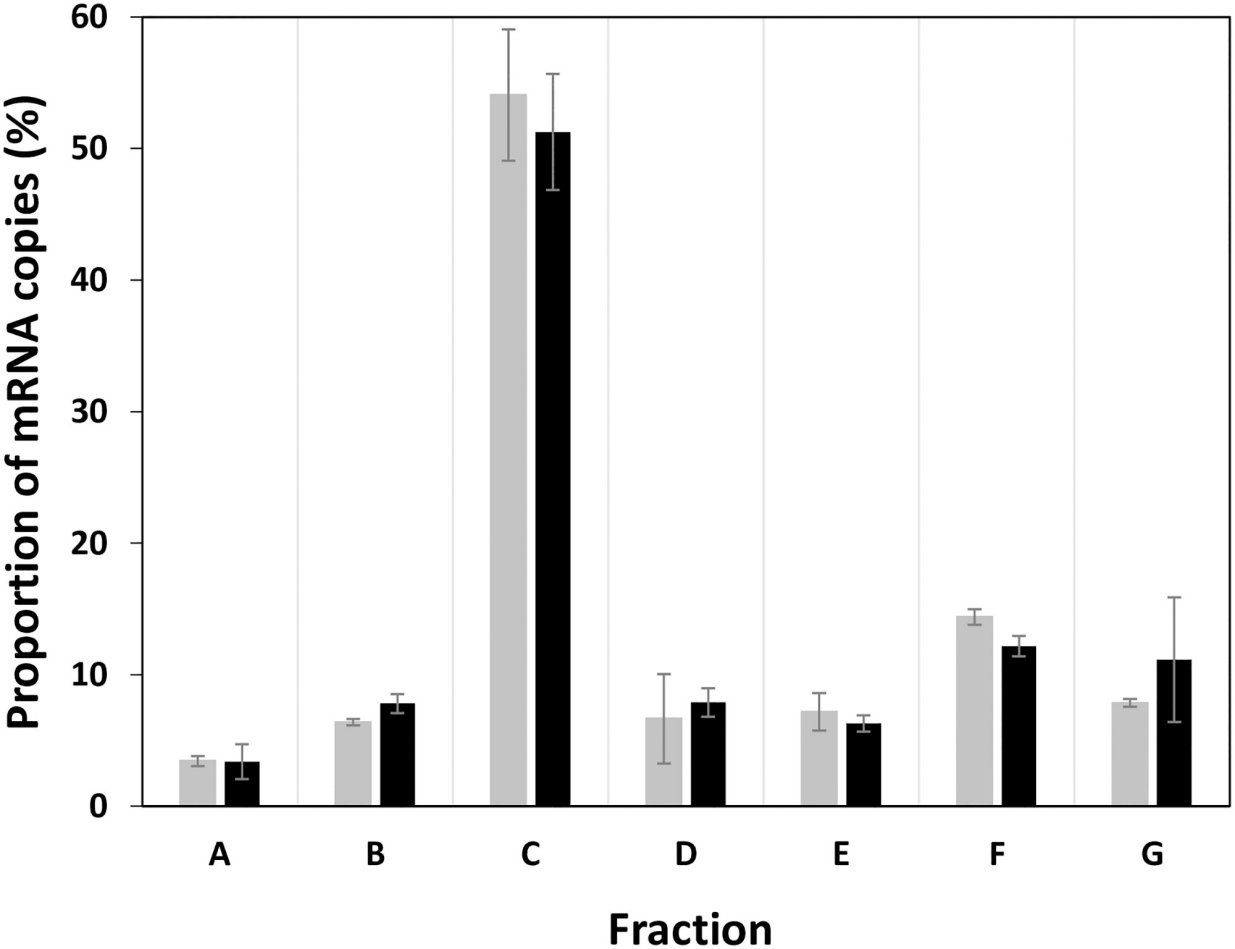
Distribution of *ihfB* mRNA copies between fractions A to G without multiplexing (grey) and after multiplexing (black). Fraction A, free mRNA not undergoing translation. Fractions B to G, mRNA bound with ascending number of ribosomes from 1 to 11. Mean values and standard deviations of two independent biological replicates are presented in the figure. Results were obtained with only MET739 cell-free extract in the non-multiplexing experiment or with MET739 cell free extract mixed with cell free extracts from five other strains in the multiplexing experiment.

To validate a multiplexing method of polysome profiling experiments, we compared the translational status of *ihfB* without multiplexing and after multiplexing with cell extracts from distinct strains. We chose to multiplex up to six cell-free extracts. The translational status without multiplexing corresponds to the polysome profiling experiment described above when only the cell-free extract of *E*. *coli* MET739 was loaded on the sucrose gradient. In the multiplexing polysome profiling experiment (Fig 1), the cell free extract of *E*. *coli* MET739 was mixed before loading on the sucrose gradient with five other cell free extracts: one from MET734 (overexpressing *cysZ*) and four from unrelated *E*. *coli* MG1655 constructs. Each cell free extract was produced from an individual culture in the exponential phase in M9 glucose medium. Quantification of *ihfB* mRNA copies in each fraction of the polysome was very reproducible within each experiment of polysome profiling (with and without multiplexing) (Fig 3). In addition, the distributions of *ihfB* mRNA copies were very similar before and after multiplexing (Fig 3). Consequently, comparable values of ribosome occupancy (96.6 ± 0.4% versus 96.6 ± 1.1%) and similar distributions of the ribosome density were obtained, the most frequent RD of the *ihfB* mRNA copies still being 1 ribosome/100 nt after multiplexing. We concluded that the multiplexing polysome profiling method did not affect the translational status observed for an mRNA and that this method can therefore be used to study translation statuses in parallel in multiple strains.

### Multiplexing polysome profiling experiment can be used to monitor the translational response between two conditions

**Fig 4:**
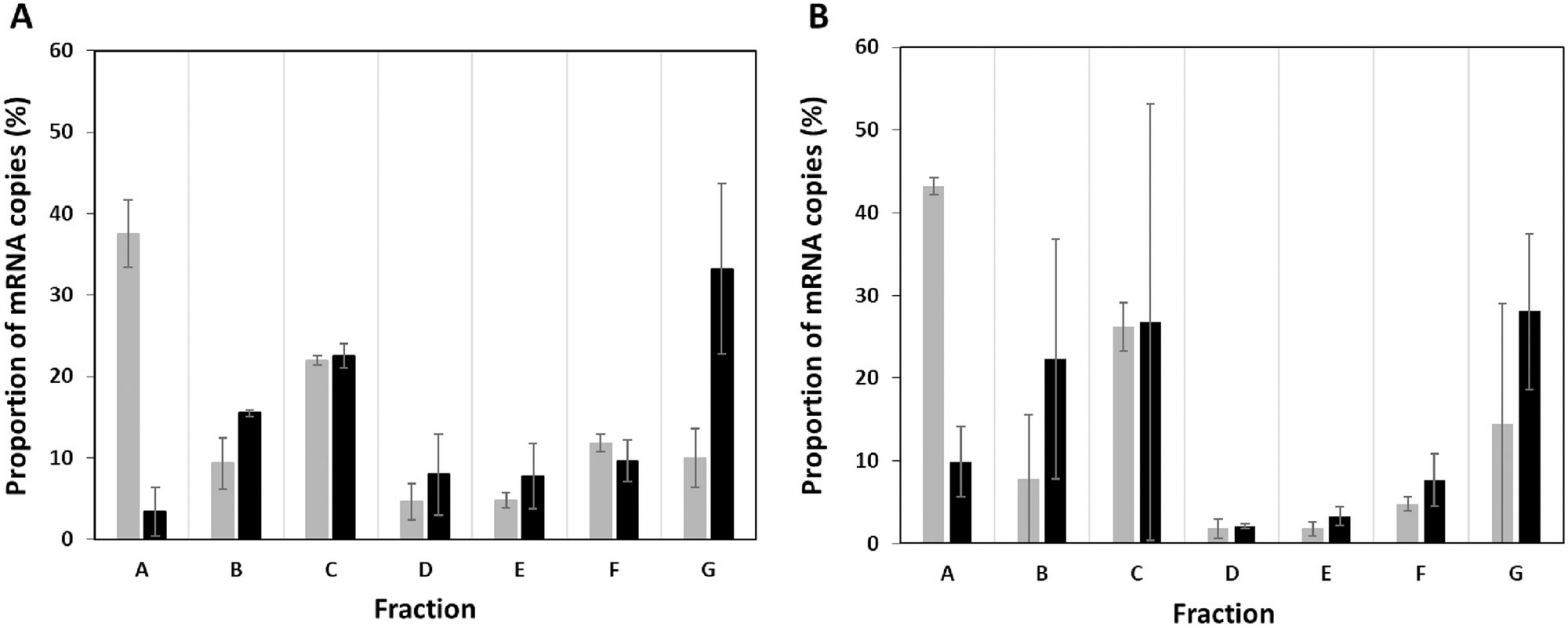
Distribution of *cysZ* mRNA copies between fractions in the two conditions: “without induction” (grey) and “with induction” (black) in (A) standard and (B) multiplexing polysome profiling experiments. Fraction A consists in free mRNA copies not undergoing translation. Fractions B to G contain mRNA copies bound with ascending numbers of ribosomes from 1 to 11. Mean values and standard deviations of two independent biological replicates are given. Results were obtained using only strain MET734 in the non-multiplexing experiment or using strain MET734 mixed with 5 other cell free extracts.

We wanted to know if the translational response of a gene between two conditions was similar using the classical non-multiplexing and our multiplexing polysome profiling methods. Using the two methods, we thus investigated the translational response of the *cysZ* gene when its mRNA level was increased. The *cysZ* gene codes for a high-affinity, high-specificity sulfate transporter that provides the sulfur source for the synthesis of cysteine [29]. Sulfate uptake by CysZ is essential for the survival of *E*. *coli* under low sulfate conditions. We focused on the *E*. *coli* MET734 strain in which the *cysZ* gene is under the transcriptional control of the arabinose inducible P_BAD_ promoter. *E*. *coli* MET734 was cultured in M9 glucose without arabinose (low *cysZ* mRNA level) and with arabinose transcriptional induction. Our measurements showed that arabinose induction led to a 27 ± 6 fold induction of *cysZ* mRNA in MET734. On the other hand, the mRNA level from the chromosomal copy of *cysZ* in MG1655 wild type was four times lower than the level observed in MET734 without arabinose induction, and about 100 times lower than in MET734 with arabinose induction. In the pool of *cysZ* mRNA copies, the part originating from the chromosomal copy of *cysZ* can thus be neglected. Therefore in the multiplexing experiments, the *cysZ* mRNAs were only assigned to strain MET734. The five other strains not carrying the *cysZ* gene on the plasmid were also cultured independently with and without arabinose induction. Four polysome profiling experiments were then performed: one non-multiplexing experiment (only the cell free extract of *E*. *coli* MET734 was loaded on sucrose gradient) and the multiplexing experiment (loading of *E*. *coli* MET734 mixed with the five other cell free extracts), with and without arabinose transcriptional induction. The translational responses of *cysZ* between low and high mRNA expression level using the two methods are shown in Fig 4.

Comparison of the distributions of *cysZ* mRNA copies using the standard (grey bars) and multiplexing method (black bars between A and B in Fig 4) showed equivalent distributions in the different fractions. This confirmed what was observed with *ihfB* that multiplexing cell free extracts did not alter the analysis of the distribution of a particular mRNA. With both the non-multiplexing and multiplexing methods, induction of *cysZ* expression led to a shift in the mRNA copies from the free mRNA fraction (fraction A) toward the more heavily ribosome bound fractions (mainly fraction G). With a high level of mRNA, ribosome occupancy increased from 62 ± 4.1% to 96 ± 3.0% without multiplexing and from 55.7 ± 0.7% to 89.6 ± 5.2% after multiplexing, reflecting the marked decrease in free mRNAs (lower proportions in fraction A). Consequently, ribosome density increased at high mRNA level to reach 1.5 ribosomes/100 nt in around 30% of the *cysZ* mRNA copies (in fraction G). These results showed that the multiplexing polysome profiling experiment allowed similar *cysZ* translational responses to higher mRNA levels: a decrease in free *cysZ* mRNA fraction and a higher ribosome load.

### Parallel characterization of translational responses to changing transcription level

**Fig 5:**
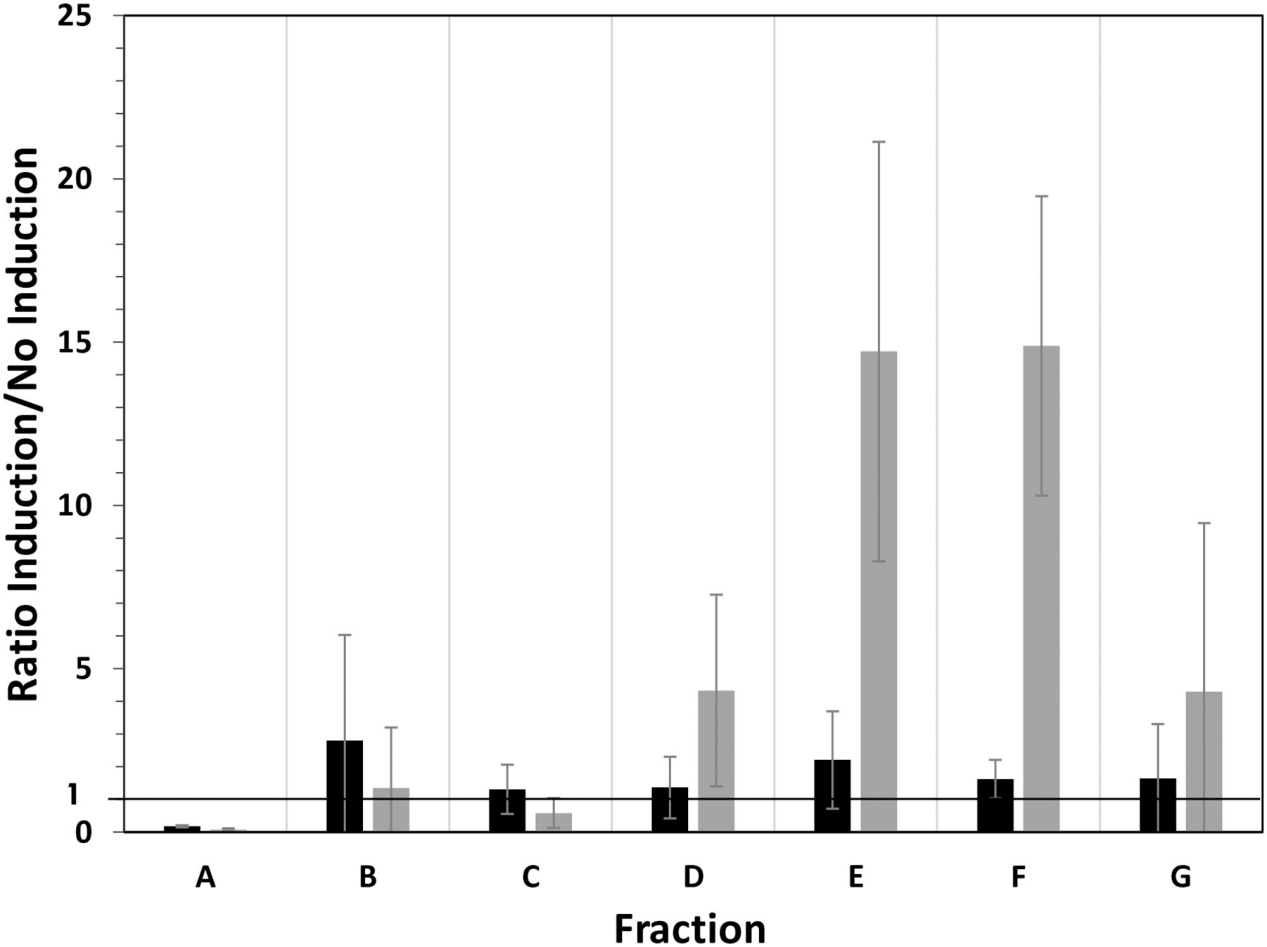
Translational response of *cysZ* (black) and *lacZ* (grey) in two mRNA expression conditions using the multiplexing polysome profiling method. For each fraction, the ratio of the proportion of mRNA copies with high mRNA levels to the proportion of mRNA copies with low mRNA levels was determined. Fraction A consists in free mRNA copies not undergoing translation while fractions B to G contain mRNA copies bound with ascending number of ribosomes from 1 to 11. Mean values and standard deviations of two independent biological replicates are given.

We used the multiplexing polysome profiling experiment to simultaneously study the translational responses of the *cysZ* and *lacZ* genes at two transcriptional levels. The *lacZ* gene codes for a β-D-galactosidase enzyme involved in lactose and other β-galactoside catabolism [30]. In *E*. *coli* MET739, the *lacZ* gene is under the transcriptional control of the arabinose inducible P_BAD_ promoter. Without arabinose, the mRNA level from the chromosomal copy of *lacZ* in MG1655 wild type was 14 times lower than the level observed in MET739. Arabinose induction led to a 50 ± 23 times higher *lacZ* mRNA level in MET739. Therefore in the multiplexing experiments, the *lacZ* mRNAs were only assigned to strain MET739. Using the two multiplexing polysome profiling experiments described in the previous section, without arabinose (low *cysZ* and *lacZ* mRNA levels) and with arabinose (high *cysZ* and *lacZ* mRNA levels), we assessed the translational responses of *cysZ* and *lacZ* to changing mRNA levels (Fig 5). We calculated the ratio of the proportion of *cysZ* and *lacZ* mRNA copies with high mRNA levels to the proportion of mRNA copies with low mRNA levels. In both genes, when the mRNA expression was induced, we observed a significant reduction of the proportion of the free mRNA copies (ratios much lower than 1 in fraction A) and therefore an increase in the more heavily ribosome-loaded mRNA copies. However the pattern of translational responses of *cysZ* and *lacZ* differed. The *cysZ* mRNA copies spread all over the ribosome loaded fractions (all fractions from B to G exhibited ratios higher than 1) whereas the *lacZ* mRNA copies shifted more preferentially to fractions E and F. The magnitude of the translational response was more than 6-fold higher for *lacZ* than for *cysZ*. These results show that the multiplexing polysome profiling experiment allowed the parallel characterization of translational responses of two genes after increasing their mRNA level. For both *cysZ* and *lacZ*, the global translational responses to increased mRNA levels consisted in a shift toward the more heavily ribosome-loaded mRNA copies but the pattern and magnitude of the responses differed.

### Specificity of the translational response

**Fig 6:**
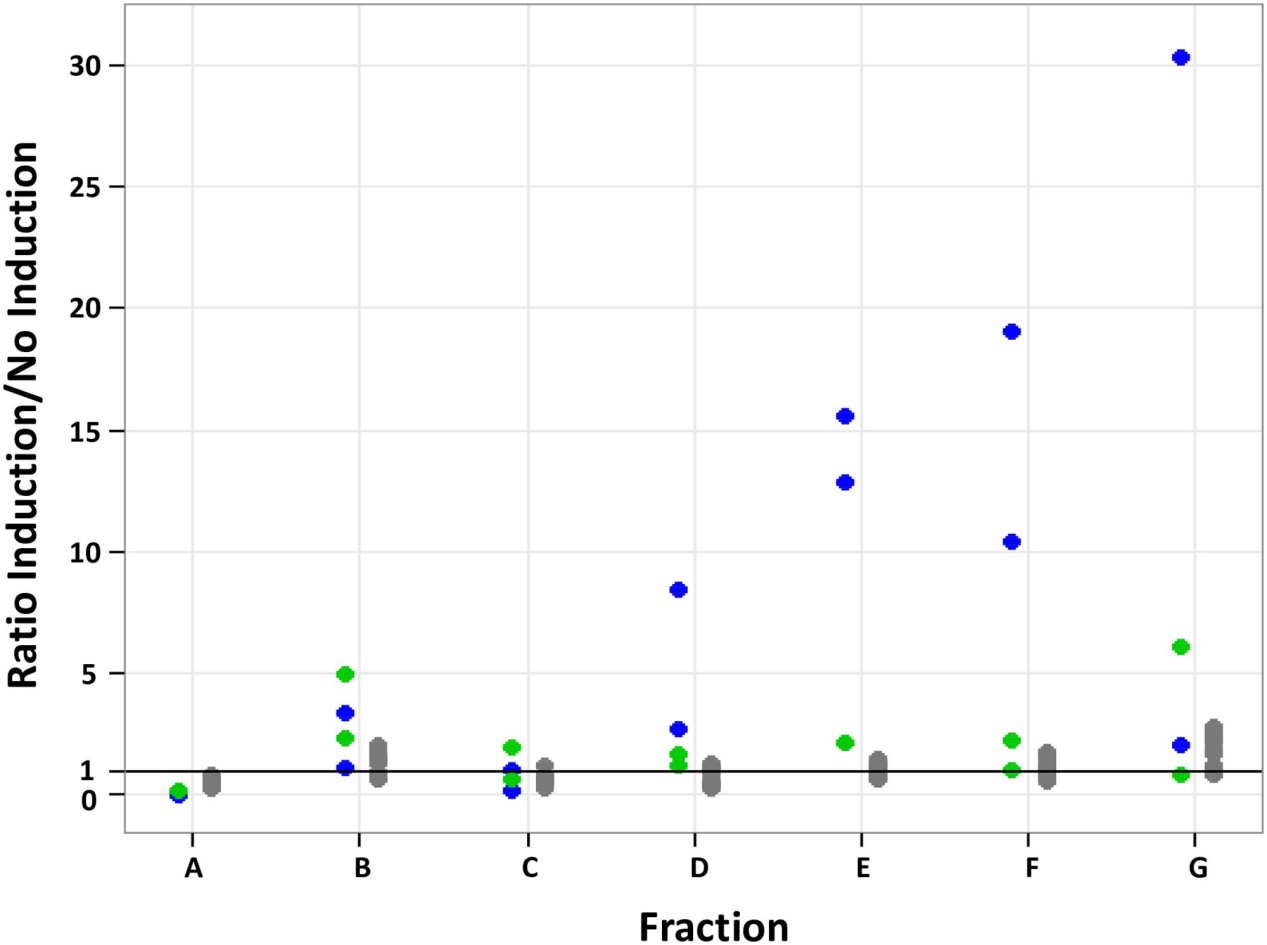
Translational response of the *cysZ* (green), *lacZ* (blue) and of 10 other unregulated genes (grey), with and without arabinose induction, using multiplexing polysome profiling experiments. For each fraction, the ratio was calculated between the proportion of mRNA copies with arabinose induction (high mRNA level) and the proportion of mRNA copies without arabinose induction (low mRNA level). Fraction A consists in free mRNA copies not undergoing translation while fractions B to G contain mRNA copies bound with ascending number of ribosomes from 1 to 11. Ratios were calculated from two independent biological replicates of each condition (2 dots are plotted for *cysZ* and *lacZ* and 20 dots for the unregulated genes in each fraction).

To check the specificity of the translational response to changing the mRNA level measured for *cysZ* and *lacZ*, we compared their responses to those of 10 chromosomally encoded genes not under the control of P_BAD_ (namely *eno*, *ihfB*, *rpsJ*, *rpsD*, *rplK*, *rplV*, *rpsL*, *trmJ*, *rnpA* and *rppH*, S1 Table). Ratios of mRNA copies proportions for the non-inducible genes were calculated in the two conditions, i.e. with and without arabinose, using the multiplexing experiments and were compared to the values of *cysZ* and *lacZ* (Fig 6). As expected, the non-inducible genes showed no significant difference in their translational response between with and without arabinose, since their mRNA level did not significantly differ between the two conditions (variations in the expression of the 10 non-inducible genes were in the range of the technical error). With arabinose, the decreases in the proportions of both *cysZ* and *lacZ* copies in fraction A were higher than those in the non-inducible genes. The increases in the proportions of *lacZ* copies in the fractions D, E and F were considerably higher than the increases in the non-inducible genes; the increase was only slight for *cysZ* in fraction B compared to the non-inducible genes. These results confirmed that the multiplexing method can be used to specifically measure the translational response of a gene after changing its mRNA level in the cell.

## Discussion

In this work, we developed and validated a multiplexing polysome profiling method to study the translation status of mRNAs and its variations in *E*. *coli*. The distribution of mRNA copies between the different polysome profiling fractions, and consequently ribosome occupancy and ribosome density, was similar using the standard non-multiplexing method and the new multiplexing method. The multiplexing method allows parallel quantification of the specific translational response to changing gene expression. In this study, we present the translational response of two genes, *cysZ* and *lacZ*, but our multiplexing approach allows simultaneous analysis of the translational response of up to six genes. In the case of *cysZ* and *lacZ*, we demonstrated a similar overall effect of the concentration of mRNA on the translation status with a higher ribosome load at higher mRNA levels but with a gene-specific pattern and magnitude of the responses. This result demonstrates co-transcriptional regulation of translation for these two genes. We hypothesize that co-transcriptional regulation of *cysZ* and *lacZ* translation contributes to physiological adaptation when cells regulate the mRNA level of genes to adapt to environmental changes (such as a low sulfate conditions [29] for *cysZ* or the availability of lactose as a carbon source for *lacZ* [30]). Further studies are now required to confirm these findings at the genome scale. Applied here to explore *E*. *coli* translation regulation, the multiplexing polysome profiling method can be expanded to any other organism. Using multiplexing saves time and effort and reduces the cost and technical bias that may result when large numbers of samples have to be handled.

In this study, we simultaneously studied the translational response of different genes to the same stimulus (i.e. their mRNA level). We used different molecular constructs in the same genetic background to trigger changes in mRNA levels. In the multiplexing experiment, we mixed cell free extracts of different strains generated in the same conditions, either without induction or with transcription induction. Another possible application of the multiplexing method is studying the translational response of one gene to different stimuli related to changes in the growth environment or to modifications in the genetic background. In this case, the multiplexing experiment will mix cell free extracts generated in different conditions. The only constraint will be that the gene of interest has to be specifically tagged (for example using barcode tagging to provide specific hybridization for qPCR) in each condition to differentiate the mRNA copies originating from each condition in the polysome profiling fractions.

Our multiplexing method will be of particular interest for the study of translation regulation at the mechanistic level. The effect of mRNA sequence-related parameters as potential regulators of translation initiation and elongation has been investigated using molecular approaches. The effect of codon usage on translation in yeast [19] or the one of 5’UTR sequence on polysome distribution in *Arabidopsis* [20] have already been investigated using polysome profiling. As these studies were limited to the characterization of only one mRNA with only three different sequence modifications, multiplexing polysome profiling experiment could easily be extended to the analysis of more genes and sequence modifications. When many samples were analyzed by the standard polysome profiling technique like in [18] to investigate the effect of eight different 5’UTR structures on the translation of a reporter mRNA in yeast, using our multiplexing method would have saved time, money and effort by analyzing a single multiplexed polysome. In the study of the effect of codon usage on translation elongation using the ribosome profiling method [31], the implementation of a complementary experiment of multiplex polysome profiling would have provided additional information on translation heterogeneity in the copies of the reporter mRNA.

Additional regulatory features of mRNA sequences on translation can be explored with the multiplexing method coupled with a high resolution PCR technique such as the TaqMan RT-PCR. Analysis of the translational response to small differences, from some nucleotides to single point mutation, in sequences suspected of being involved in translation regulation (like sequence motif (conserved pattern [7] and SD-like motif [32]), secondary structure [33,34] for the binding sequences of regulatory ncRNA [35] and proteins (for instance CsrA [36]) could be performed more easily and quickly using the multiplexing method coupled with highly specific TaqMan probes. The technique could be also used to study the translational effect of natural single nucleotide polymorphism, for example between different alleles [37,38], to tackle the long-term evolution of translation regulation.

In conclusion, the multiplexing polysome profiling method is a low scale method mainly useful to study translation of (i) several reporter mRNAs (with different expression level, 5’and 3’ UTR sequences or coding sequence), (ii) endogenous genes in a strain when they have been previously tagged with specific artificial sequences and (iii) different natural alleles of a gene found in closely related strains or species. Furthermore, at the genome-wide scale, this method coupled with RNA sequencing can also be used when mixing microorganisms with distinct genetic backgrounds. In this case, the possibility to assign the sequenced reads to the specific genes of each microorganism will allow the translation of these genes to be studied. The multiplexing method could open the way for “metatranslatomics” analyses.

## Supporting information

S1 Fig Ratios of the 23S rRNA and 16S rRNA amounts in sub-fractions of the polysome profiling experiment (grey line). The ratio of 16S/23S rRNA amounts is in blue and the ratio of 23S/16S rRNA amounts is in brown. The sub-fractions are delimited by vertical black lines.
(TIF)

S1 Table Sequence of qPCR primers used to quantify 12 endogenous genes of *E*. *coli* MG1655 and four ERCC RNA spike-ins. (DOCX)
