# Supplementary figures and images for: Multiplexing polysome profiling experiments to study translation in Escherichia coli
Source: PLoS One. 2019 Feb 19;14(2):e0212297. doi: 10.1371/journal.pone.0212297 (PMC6380557; doi:10.1371/journal.pone.0212297)

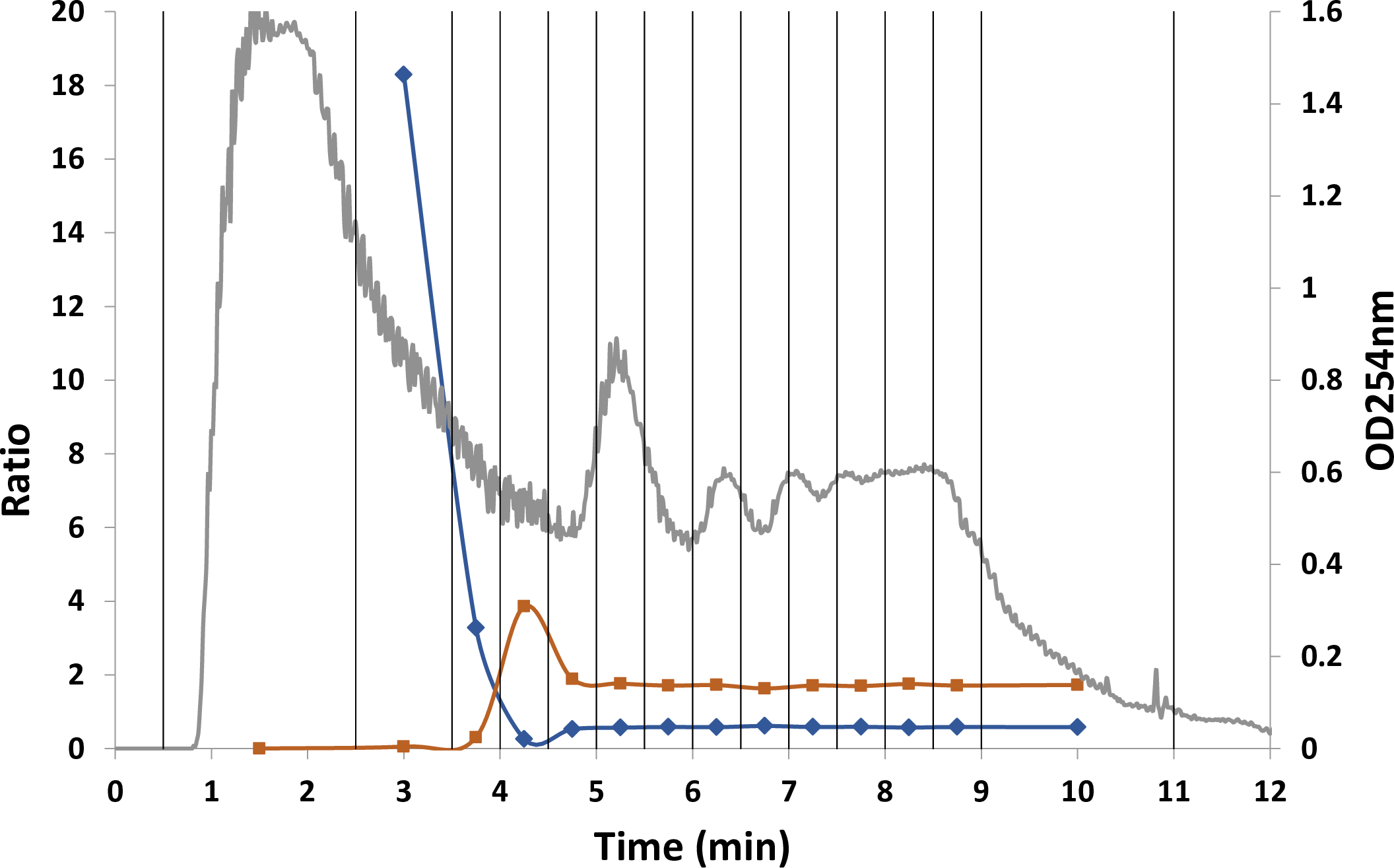

Supplement: S1 Fig — The ratio of 16S/23S rRNA amounts is in blue and the ratio of 23S/16S rRNA amounts is in brown. The sub-fractions are delimited by vertical black lines. (TIF) [file pone.0212297.s001.tif]
