# Supplementary material for: Multiplexing polysome profiling experiments to study translation in Escherichia coli
Source: PLoS One. 2019 Feb 19;14(2):e0212297. doi: 10.1371/journal.pone.0212297 (PMC6380557; doi:10.1371/journal.pone.0212297)
Supplement: S1 Table — (DOCX) [file pone.0212297.s002.docx]

| Gene | Function | Forward primer | Reverse primer |
| --- | --- | --- | --- |
| *cysZ* | Sulfate transporter | CATCATTCACATCTGCCCCACG | GCGCCCCCCATCAACAAAATAT |
| *lacZ* | β-D-galactosidase |  |  |
| *pair_1* |  | TCCGTGACGTCTCGTTGCTGC | TCACGCAACTCGCCGCACAT |
| *pair_2* |  | CCCGCATCTGACCACCAGCG | CAGCGGCGTCAGCAGTTGTT |
| *pair_3* |  | GTCGTGACTGGGAAAACCCTGG | AACTGTTGGGAAGGGCGATCG |
| *pair_4* |  | AACAACTTTAACGCCGTGCGCT | CACCATGCCGTGGGTTTCAATA |
| *pair_5* |  | CAGCTGGCGCAGGTAGCAGAG | GGCAGATCCCAGCGGTCAAA |
| *ihfB* | Integration host factor | GCCAAGACGGTTGAAGATGC | CAAAGAGAAACTGCCGAAACC |
| *trmJ* | Methyltransferase | TCGAATTGTGCTGGTGGAGACG | AATCGCCTGGGAGTCGGGTT |
| *eno* | Enolase | GTCGTGAAATCATCGACTCC | CAGCTTTGGTTACGCCTTTA |
| *rppH* | Pyrophosphohydrolase | AGAAGTAGGATTAAGCCGCA | GTCAAACTCTGGTGTACTGC |
| *rnpA* | RNase P protein component | AACGCAATCGGATTAAACGT | GGCGCCATAATTTTTCCAAC |
| *rpsD* | 30S ribosomal subunit protein | CGTGAGGGCACCGACTTATTC | AGTCAGACAGACGCGGTTTACG |
| *rpsJ* | 30S ribosomal subunit protein | CGTACTCACTTGCGTCTGGTTG | AGGCTGATCTGCACGTCTACAC |
| *rpsL* | 30S ribosomal subunit protein | CATCGGTGGTGAAGGTCACAAC | CTTTAACGCCGGAGCAGTCAAG |
| *rplK* | 50S ribosomal subunit protein | AGCGGCTGGTATCAAGTCTGG | GTCATGTCGGCAGCTTTGGTC |
| *rplV* | 50S ribosomal subunit protein | CGCGGTAAGAAAGTGTCGCAG | CAGCGCCATCGTTGTGTTCAG |
| ERCC 130 | RNA spike-ins | GTGAAGATGATTGACCGCACGC | TGCATATTGCAGCTGAGCCAGC |
| ERCC 002 | RNA spike-ins | CCGTCGGCTGATCGTGGTTT | CGACCGTACAGCTCTGGAACCC |
| ERCC 074 | RNA spike-ins | GCCTTGGTAGGGATAGATAGCCACC | CTGGGGTTATGAGTAGGGATGAGCA |
| ERCC 096 | RNA spike-ins | CGTAACCAAACATGCACAGCGG | TCGCGTCATCGATCCGGGT |

**S1 Table:** Sequence of qPCR primers used to quantify 12 endogenous genes of *E. coli* MG1655 and four ERCC RNA spike-ins.
